# Near-Death-Like Experiences without Life-Threatening Conditions or Brain Disorders: A Hypothesis from a Case Report

**DOI:** 10.3389/fpsyg.2012.00490

**Published:** 2012-11-15

**Authors:** Enrico Facco, Christian Agrillo

**Affiliations:** ^1^Department of Neurosciences, University of PadovaPadova, Italy; ^2^Italian Center for Clinical and Experimental HypnosisTorino, Italy; ^3^Department of General Psychology, University of PadovaPadova, Italy

**Keywords:** death studies, body–mind problem, consciousness

## Abstract

Near-death experiences (NDEs) are profound psychic experiences commonly occurring in life-threatening conditions. They include feeling a sense of peace, of seeing a bright light, encountering deceased relatives or religious figures, and of transcending space and time. To explain them, it has been suggested that they stem from brain disorders and/or psychological reactions to approaching death, a sort of wishful thinking in response to the perceived threat. This is a report on a case with most of the features typical of NDEs except that it occurred entirely without any life-threatening conditions. This evidence is theoretically incompatible with either of the above hypotheses, suggesting that a broader interpretation of the phenomenon is needed.

## Introduction

Near-death experiences (NDEs) are an intriguing neuroscientific topic with profound epistemological implications and an increasing number of publications (Greyson, 2007, 2010; Griffith, 2009; Metzinger, 2009; Terhune, 2009; Facco, 2010; Agrillo, 2011; Mobbs and Watt, 2011). NDEs may include several perceptions, such as seeing a bright light, moving through a tunnel, positive emotions, meeting deceased relatives, communicating with a light, entering a new domain, reaching a point of no return, reviewing one’s life, and out-of-body experiences (OBEs). Their incidence has probably increased due to advances in resuscitation methods. NDEs are reported in all cultures (Belanti et al., 2008) and, although a culture-related incidence of some particular features (i.e., “life review” and tunnel perception) has been described (Kellehear, 1993), the core experience seems to be much the same across cultures (Greyson et al., 2009). Both psychological and physiological interpretations have been suggested (Table 1). Psychological interpretations are based on the “expectation hypothesis” and consider NDEs the product of an altered mental state induced by life-threatening conditions (Blackmore and Troscianko, 1988; Appleby, 1989; Britton and Bootzin, 2004), their phenomenology presumably stemming from the projection of beliefs and expectations of an afterlife. It is still not clear, however, how people suddenly finding themselves in critical conditions (e.g., cardiac arrest) might be aware of being near-death and have time enough to develop complex scenarios to suit their wishes. Spiritual and religious experiences are global human phenomena that have always occurred down the ages, while the transcultural features of NDEs (whatever their meaning) appear to be an archetypal expression of the human mind. This being so, there could be no cultures completely free of any form of religion, enabling us to verify the expectation hypothesis: even in countries that have abolished all forms of creed (like the USSR in the twentieth century), many people retain a deep religiosity hidden in their psyche.

**Table 1:** **Main reductionist models for explaining NDEs**.

Neurobiological hypotheses	Psychological hypotheses
Altered blood gas level	Expectation
• Hypoxia	Depersonalization
• Anoxia	Derealization
• Hypercapnia	Dissociation
Periphery-to-fovea retinal ischemia	Personality
Neurological factors	Birth model
• Epilepsy	False memories
• Excitotoxic damage (glutamate)	
• Neurotransmitter imbalance	
• REM-sleep intrusion	
• Temporal lobe disorders	
• Multisensory breakdown – right angular gyrus dysfunction	
Pharmacological factors	
• Opioids	
• Steroids	
• Ketamine	
• Hallucinogens	

Other psychological theories focus on several possible aspects (see Greyson et al., 2009; van Lommel, 2010 for a full review of these factors), including: (a) depersonalization, i.e., NDEs would be a sort of profound depersonalization with a subsequent loss of identity; (b) a feeling of detachment and unreality; (c) dissociation (a defense mechanism to evade a frightening reality, such as a life-threatening event, which involves a disruption of identity, memory, and consciousness); (d) personality factors (even though attempts to identify personality traits correlating with NDEs have so far remained inconclusive); (e) memory of birth, according to which a dark tunnel, a bright light, and going toward a new landscape would be a recollection of the experience of birth. False memories have also been suggested, i.e., a sort of attempt made by the mind to retrospectively fill the gap after a period of unconsciousness (French, 2001). Braithwaite (2008) made the point that the brain is constantly trying to make sense of the information it receives so as to preserve a rapid and coherent interpretation of this information, so NDEs could be seen as patients’ attempts to make sense of their confusing experience (Mobbs and Watt, 2011).

Neurobiological hypotheses consider NDEs as a by-product of brain disorders. Altered blood gas levels (i.e., hypoxia or hypercapnia) have been suggested by some authors to have a major role in producing hallucinations, such as tunnel vision and bright lights (Blackmore, 1996); and an abnormal electrical activity (REM intrusions and epilepsy in the limbic system and temporal lobes) has been invoked to explain life memory flashbacks (Britton and Bootzin, 2004; Nelson et al., 2006). It has also been postulated that medication, and neurochemical reactions in general, could affect the occurrence of NDEs (Mobbs and Watt, 2011), but delirium in critical care patients (Facco and Rupolo, 2001; Arend and Christensen, 2009), which is the well-known primary effect of brain dysfunction and/or drugs, is entirely different from NDEs. Jansen (1990, 1991) claimed that NDEs might be due to an involvement of NMDA receptors in neurocritical patients, and this Author also suggested a link between NDEs and hallucinogens such as ketamine (see Facco, 2010, for a review on the subject). On the other hand, single, witnessed NDE cases (van Lommel et al., 2001) challenge the reductionist view of human consciousness, pointing to the awkward idea of a possible transient dissociation between body and mind.

Some features of NDEs have occasionally been observed in people who are not in life-threatening conditions, in whom there can be no expectations about the hereafter or neurotransmitter disorders involved. These cases might be related to psychological disorders, such as autoscopy, but OBEs have distinct features and do not correspond to the classical definition of autoscopy (Facco, 2010). The main neurophysiological mechanisms behind them have been elucidated and they can be at least partially reproduced in healthy subjects (Ehrsson, 2007), while memory flashbacks following temporal lobe stimulation had already been reported in the mid-twentieth century by Penfield (1955).

To date, little attention has been paid to ascertaining whether some features of NDE are exclusive to life-threatening conditions. Gabbard and Twemlow (1991) compared OBEs occurring in life-threatening versus non-critical conditions, failing to find any feature exclusive to the former. Some NDE components were more likely in individuals who sensed that death was near (regardless of whether their conditions were genuinely life-threatening or only perceived as such), suggesting a role of expectancy (Gabbard and Twemlow, 1991), though this can only apply to cases in which consciousness is preserved.

As far as we know, there have been few reports of profound NDE-like experiences in people who were not in life-threatening conditions, but their repeated occurrence would challenge both the neurobiological and the psychological hypotheses advanced to explain the pathophysiology of NDEs. Even their name and definition would have to be reconsidered because it would mean that these psychic experiences might potentially be perceivable in everyday life, and their meaning would still be unknown when they occur unrelated to any psychological disturbances. In this paper, we report on the case of a person who had a near-death-like experience in healthy, physiological conditions.

## Case Report

The subject was an electrotechnician who came under our observation because he was trying to understand the meaning of a strange experience he had had a few years earlier, when he was divorcing from his wife.

He told the following story: “*I was spending my summer holidays in the mountains with my four-year-old child. I had recently separated from my wife; it was a difficult time. One evening, while I was in the room where we were staying, I suddenly saw a great white light. It was not dazzling, but its whiteness was unnatural, I mean it did not seem to be like the white light from natural or artificial sources we know, nor did it come in from outside. Then, some balls of light appeared; I did not count them, but there were perhaps five or six, and they could have been about 1.5 meters in diameter. These balls were translucent with the same color as the light, but less transparent and thicker, though I noticed that they did not cast any shadow. At the time, I had a profound feeling as if all the beings of the world were within me and, at the same, I felt as if I were within them. The source of light was ellipsoid. It was Love and Joy, and I felt a sort of stream through me. I use the term ‘stream’, but it was not so clearly definable. I cannot use the term ‘wind’, because wind comes from outside, while I felt this stream inside me. I was so enraptured that I had stopped breathing. I was fully lucid, however, and realized that I was not breathing, so I started breathing again, but my breathing disturbed the vision and, after a few breaths, it vanished.”*

The subject reported that he had rejected any form of religion by that time in his life, as a reaction to having gone to a Catholic college for a year during his adolescence. His experience had surprised him because it was different from anything he could imagine. The experience was very strong and led to a profound transformation. As a result of it, he overcame any fear of death, which he no longer saw as a negative event, but as an intrinsic, inseparable part of life, and of an ineffable divine beyond any conventional concept. Now he is happy with his new wife, but he is continuing to study in search of the meaning of his experience, and of life itself. Far from being fideistic, his stance is rational and agnostic.

The score on the Greyson scale (Greyson, 1983) was 16 and consisted of the following items: (a) vision of a supernatural light; (b) complete loss of the sense of time; (c) peacefulness; (d) deep joy; (e) empathic fusion with the whole world; (f) clear perception of a reality beyond the ordinary world; (g) understanding everything about the universe; and (h) encounter with entities.

After his experience, he immediately felt the need to write down his thoughts in the form of a poem, symbolizing the perception of immanence of an impersonal divine in the world and its loss with the exclusive use of logic, reasoning, and erudition. He still feels that he is unable to really explain his deep experience, with attendant disappointment; he thinks this is partly due to his lack of a humanistic background.

## Discussion

This was a case of a rather profound empathic–mystic experience. While the score on the Greyson Scale confirms its intensity and likeness to an NDE, no cerebral, or psychological disorders, nor any drug-related effects can explain its occurrence, challenging any reductionist or mechanistic interpretation of the phenomenon. The subject had no history of psychological disorders, use of psychotropic drugs, or substance abuse. On the whole, his life could be described as completely normal. While he has always been aware of the oddity of his experience, it has never led him to strike a fideistic pose; instead, he has continued to have all his doubts and tried to understand the meaning of the experience.

The present account is consistent with a growing body of literature on NDEs occurring in the absence of life-threatening conditions (Holden et al., 2009). van Lommel (2010) summarized some of the most often recurring circumstances that might prompt NDEs in subjects with no brain function disorders, including serious (but not immediately life-threatening) conditions, isolation, depression, existential crisis, meditation, and the so-called “fear-death experiences”. Another circumstance was described by Moody and Perry (2010), who reported “shared death experiences” in healthy people attending the moment of death of a close relative.

Greyson et al. (2009) made the point that, when the recurrent features of NDEs are examined one by one, they might be partially explained by psychobiological factors. In contrast, when these features occur together, as in classic NDEs, such interpretations appear strained. Indeed, there would seem to be good reason to propose a broader interpretation of NDEs than the one encompassed in the reductionist interpretations alone (Facco and Agrillo, 2012). In a prospective multicenter study on individuals experiencing cardiac arrest, van Lommel et al. (2001) found that only 12% had a core NDE. These Authors claimed that most of their patients would have had an NDE if these experiences were due merely to physiological factors, thus calling the meaning generally attributed to them into question. In their sample, moreover, neither the individual’s physical conditions (duration of the cardiac arrest, duration of unconsciousness, intubation, induced cardiac arrest, medication), nor their psychological state (fear of death, religion, prior knowledge of NDEs) correlated statistically with the incidence of NDEs, suggesting that psychobiological factors alone are not enough to explain the occurrence of NDEs.

The perception of undefined entities (not belonging to the iconography of the subject’s religion) and figures other than deceased people known to the subject would seem to be incompatible with the expectation hypothesis. NDEs have also been reported in small children presumably not old enough to have philosophical concepts or expectations and wishes concerning the hereafter (Morse et al., 1986). Patients actually nearing death report experiencing a more enhanced cognitive function than others whose illness or injury does not put their life at risk (Owens et al., 1990). Here again, this would hardly appear to be compatible with the reductionist idea of a close dependence of the mind’s functioning on the brain, an impairment of the latter implying a parallel cognitive function impairment.

The time is probably ripe for a reappraisal of the very concept of NDEs as encompassing spiritual experiences as a whole. Given the huge number and variety of transcendent experiences reported by Rankin (2008), there seems to be good reason to do so. We defined our subject’s experience as “NDE-like” to emphasize both its similarities and differences vis-à-vis true NDEs. In this regard, it is worth noting that advances in our knowledge and classification of events is context-sensitive, and that is why transcendent experiences seen in the field of emergency medicine have prompted physicians to name them NDEs and interpret them mainly from a neurobiological perspective (the experiences reported by terminal patients have been dubbed “deathbed visions” or “end of life experiences”), while psychologists, philosophers, and religious people are more inclined to see them as spiritual and religious experiences, observing them outside the context of clinical disease. This has given rise to a number of diverse phenomena being defined differently in the context of different disciplines, which have several features in common as well as phenomenological differences. Our patient, whose case belongs to the field of spiritual experiences, sought the authors’ opinion after hearing about the latter’s interest in NDEs.

All non-ordinary activities of consciousness, in states of brain dysfunction, psychotic disorders, hypnosis, meditation, ecstatic and mystical states, and those induced by psychotropic drugs, have been gathered under the umbrella term of “altered state of consciousness” (ASC) in the science of psychobiology (Vaitl et al., 2005). This term ASC is questionable, however, at least to some degree (Facco, in press), because it suggests *a priori* the idea of a dysfunction, while some of the states it is meant to describe (e.g., meditation and hypnosis) imply no such abnormality.

In short, there is some sort of link between the phenomenology of NDEs, mystic experiences, the visions of prophets and apostles in the Holy Scriptures, inspiration in poetry, art and music, meditation, hypnosis, neurological diseases (such as temporal lobe epilepsy), psychiatric disorders, and the effects of hallucinogens (see Facco, 2010, as a review on the subject). There may also be a *trait d’union* between NDEs, hypnosis, and meditation (Otani, 2003; Facco, in press). Similarly, in the collective imagination there is a link between genius and madness. The dominant mechanistic and reductionist approach in biomedicine focuses on diseases and mainly adopts a statistical concept of normality, so it may also risk misunderstanding the nature of “awkward” expressions of the mind, taking for a disorder something that is not, especially in the field of mind expressions and psychiatry (Wakefield, 2010).

The concept of the plausibility of thoughts and experiences has deep social and epistemological implications too, and depends on the so-called *Zeitgeist* (spirit of the time): a vision or premonitory dream would have been considered normal and even welcome in the Ancient World, whereas from our modern-day materialistic viewpoint, their oddity makes us interpret them *a priori* as false or possibly related to brain dysfunctions or psychiatric disorders, thus returning us to the eternal philosophical dilemma of truth and the mind–brain–world relationship.

A variety of spiritual experiences correlate with the activation of a large fronto-parieto-temporal network where the activity of the left and right parietal systems seems to play a crucial part in transcendence (Urgesi et al., 2010). Religious thinking seems to be associated with brain regions relating to emotion, self-representation, and cognitive conflict, while thinking about ordinary things is more closely related to memory networks (Harris et al., 2009). The emerging neuropsychology of spirituality and religion thus promises to improve our knowledge of its neurocorrelates and their link with other experiences, such as those induced by hypnosis, meditation, and psychotropic drugs, helping us to go beyond the conventional approach biased by context and a prejudicial idea of dysfunction.

The “awkward” features of our subject’s experience cannot simply be passed off as symptoms of a psychiatric disorder; quite the reverse, the experience yielded positive cognitive effects. The poem he wrote concisely indicates the same path to enlightenment as described in Taoism, Buddhism, and Sufism. These disciplines claim the illusoriness of our ordinary perception of the world and the need to go beyond the limits of our ego and its logical, conceptual, and dualistic thinking to remove the mask of illusion, by means of a thorough epistemological and introspective understanding of the mind’s physiology (Humphreys, 1951; Sukuki, 1958; Izutsu, 2010). It is surprising that our subject should have been so in tune with Eastern philosophies, since he had no philosophical background and had rejected Catholicism (the only religion he had known, in a stiffly dogmatic version that he drastically refused).

The transcendental tone of such experiences, belonging to the realm of the ineffable, is a feature common to our case, to NDEs, and to mystic experiences, like the OBE-like experiences reported by Saint Paul (2 Cor. 12, 1–4). The positive nature of their content and their transforming impact also seem hard to attribute to a mere result of neurological or psychiatric impairment.

The content of our subject’s experience also closely resembles the empathogenic and entheogenic component of the effects of hallucinogens (Nichols, 2004), but there is no chance of such drugs having a role in our case. On the whole, hallucinogens generate a variety of experiences depending on several factors, including the subject’s personality, environment, and the reasons for using the drug, while NDEs have a stable frame in all cultures (Belanti et al., 2008; Facco, 2010). It would be worth reconsidering the role of hallucinogens across the entire history of humanity, right from prehistoric times, through the Eleusinian Mysteries in Greece, to the shamanic cultures and native religions, where such substances have close links with rituals and spirituality – a usage that is poles apart from the drug abuse of our time in our culture. The closest connection between NDEs and the products of hallucinogen use reportedly lies in the use of iboga in the Bwiti religion of Gabon, where this hallucinogen is used in a complex rite of passage that intentionally simulates a NDE (Strubelt and Maas, 2008). The image of entities in the form of balls of light as described in our case seems curiously to exist in Mexican shamanism, at least as in the descriptions in Castaneda’s (1997) fiction.

Near-death-like experiences could seem to derive from role transition too (Gabbard and Twemlow, 1991). In our case, the divorce was the only detectable factor that might have been related to the experience. We might speculate that such an event involves a subject in a profound reflection on the existence and might facilitate non-ordinary activities of consciousness and trigger this type of experience. NDE-like experiences can be induced in hypnosis as well, and have been proposed as a method for facilitating both first- and second-order changes in psychotherapy (Schenk, 1999).

The lack of any organic, pharmacological, or psychiatric factors sorely tries any reductionist interpretation of the nature of NDE-like experiences and supports the need to reappraise these phenomena, taking a broader approach. Psychological factors, such as expectations or fears of death, are likely to have a limited role too. For the time being, we can only suggest the following speculations.

**Figure 1:**
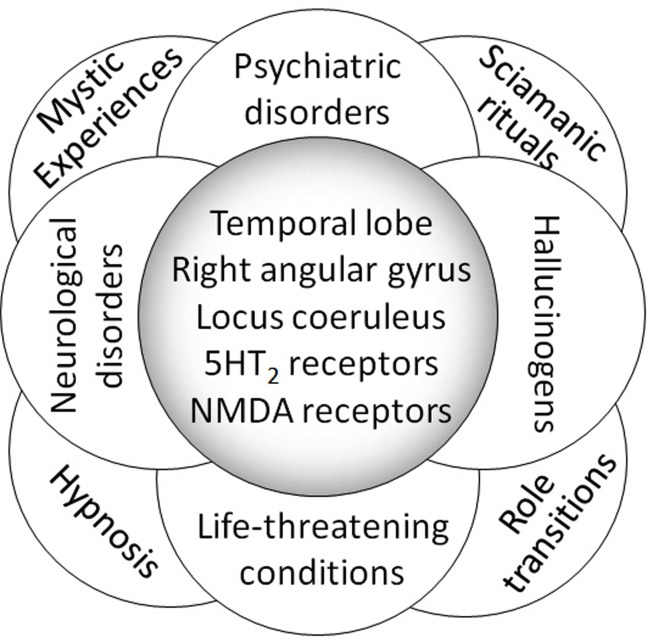
**Physiological and pathological contexts giving rise to NDEs, NDE-like experiences, and non-ordinary experiences of transcendental tone**. These experiences might involve the same brain areas and neurotransmitters (an example of which is given here), and their dysfunctions seem to be understood rather better than their physiology. The latter belongs to the world of spirituality and the highest expressions of the human mind and culture, and the meaning of these experiences cannot be reduced *a priori* to a mere brain circuitry dysfunction simply because they look awkward from a materialistic perspective.

a) Apart from any dysfunctions and lesions responsible for generating symptoms, all brain areas and neurotransmitters potentially involved in these experiences may have an as yet misunderstood physiology that could provide a common terrain for all non-pathological visions reported in all cultures and at all times of human history, spirituality, and art.
b) Hallucinogens and psychotropic drugs can generate the so-called ASC, by activating these areas and probably also complex and as yet little known brain circuits; a broad range of experiences may occur as a result, the content and meaning of which are far from being just a by-product of a given substance, but also depend on the subject’s mind, with profound cultural implications.
c) Any agent capable of triggering these circuits might yield such experiences. This may be the case of life-threatening situations (both organically and psychologically), in psychiatric and neurological disorders, but also in hypnosis and meditation, role transitions and, more generally, all deep existential crises (Figure 1).
d) If this is so, then the term ASC, which has been applied to both hallucinogens and physiological activities of the mind (i.e., hypnosis and meditation), might become questionable: *altered* already suggests an idea of abnormality, a matter for the psychiatrist, while *state* is unsuitable because consciousness is never a state, but rather a ceaseless processing unit. The term *non-ordinary activity* *of consciousness* would be semantically more appropriate.
e) The reductionist approach, by aiming to detect which brain areas and neurotransmitters are involved, can continue to make an essential contribution to our scientific knowledge, but it is blind to meanings and values, and the impact of experiences on real life, which is the other, unavoidable, subjective side of the coin. Assuming from the start that these awkward experiences are only worthless epiphenomena of brain circuitry may be misleading because all products of the mind are of the same nature, including science itself, while their conceivability and plausibility depend on the paradigm adopted and, more generally, on the *Zeitgeist*.

In conclusion, our case of a very uncommon and profound spiritual NDE-like experience in normal conditions of mental and physical health challenges the conventional reductionist medical interpretations of NDEs because it combined oddity with normal function, and because it revealed cognitively positive effects, which make it no less than “normal.” It is worth emphasizing that the Greyson scale may not be a conclusive arbiter of NDEs. Other transcendent experiences that have nothing to do with NDEs might rate highly on the Greyson scale. The similarities between our subject’s experience and NDEs suggest that a link exists between transcendent experiences and NDEs, and that its content has an archetypal nature that remains to be fully elucidated. Perhaps all the relevant subjective phenomena belonging to the suspect sphere of transcendence, spirituality, and possibly also the highest expressions of the mind should be reconsidered from a “physiological” perspective, including their mechanisms, content, and meanings, without mechanistic prejudices.

## Conflict of Interest Statement

The authors declare that the research was conducted in the absence of any commercial or financial relationships that could be construed as a potential conflict of interest.
